# General practitioner knowledge, skills and attitudes to eating disorders

**DOI:** 10.1186/1753-6561-9-S1-A33

**Published:** 2015-01-14

**Authors:** S Doherty, L McNamee

**Affiliations:** 1 Department of Psychology, Royal College of Surgeons in Ireland, 123 St. Stephen’s Green, Dublin 2, Ireland

## Background

Given that general practitioners are perfectly placed to detect eating disorders this summer research study aimed to examine general practitioners' knowledge, skills and attitudes towards eating disorders. The study aimed to compile a national picture of the diagnosis, referral practices, and management of eating disorders in primary care in Ireland.

## Methods

An online survey consisting of 20 questions previously used in an American study was emailed to Irish general practitioners (GPs) of which 226 emails were read. The study remained open over a three week period. The email addresses were obtained via the Irish Medical Directory and through phone calls to surgeries. Ethical approval was granted by RCSI.

## Results

Response rate was 22%, similar to previous studies in this field. More than one third of general practitioners reported they don\'t have the skills to treat an eating disorder (ED). More than a quarter reported there were no resources available to them for treating eating disorders and 38% had missed an ED diagnosis with a patient who was subsequently discovered to have an eating disorder. A total of 60% of respondents felt uniformed regarding how to conduct an ED screening. 54% said they didn\'t know how best to talk to an ED patient about weight management. Almost half responded that they felt uninformed about what local resources were available to them

## Conclusions

Eating disorders are currently underdiagnosed in primary care. Further information about prevalence rates and additional training opportunities are desired by GPs in the area of eating disorders. Improved referral facilities are required especially outside of Dublin. A nationwide study of GPs is necessary as a follow up to this pilot study in order to get a complete picture of eating disorders in Ireland.

